# Fractalkine and CX3CR1 Levels in Gingivitis and Stage 3 Periodontitis Patients Following Non-Surgical Periodontal Therapy: A Prospective Clinical Study

**DOI:** 10.3390/jcm15134922

**Published:** 2026-06-24

**Authors:** Zeynep Pinar Keles Yucel, Bahattin Avci

**Affiliations:** 1 Department of Periodontology, Faculty of Dentistry, Giresun University, 28200 Giresun, Turkey; 2 Department of Biochemistry, Faculty of Medicine, Ondokuz Mayis University, 55270 Samsun, Turkey

**Keywords:** CX3CL1, CX3CR1, fractalkine, gingival crevicular fluid, gingivitis, periodontitis, periodontal treatment

## Abstract

**Background/Objectives**: This study aimed to evaluate the gingival crevicular fluid (GCF) levels of fractalkine/CX3CL1 and CX3CR1 in patients with gingivitis and periodontitis before and after non-surgical periodontal therapy. **Methods**: A total of 90 individuals comprising 30 with stage 3 periodontitis, 30 with gingivitis, and 30 periodontally healthy, were enrolled in the study. Gingivitis and periodontitis patients underwent non-surgical periodontal treatment. GCF samples were collected at baseline and at 1 and 3 months after treatment. CX3CL1 and CX3CR1 were measured by an ELISA analysis. **Results**: GCF CX3CL1 and CX3CR1 were significantly elevated in patients with periodontitis and gingivitis compared to healthy controls (*p* < 0.001). The periodontitis patients also showed higher GCF levels of CX3CL1 and CX3CR1 than those with gingivitis (*p* < 0.001). Significant decreases in GCF CX3CL1 and CX3CR1 were detected at 1 month after periodontal treatment compared to baseline values in both the gingivitis and periodontitis patients (*p* < 0.001). Moreover, the periodontitis patients exhibited significant decreases in both CX3CL1 and CX3CR1 levels at 3 months post-treatment compared to 1 month (*p* < 0.001), whereas no significant changes were observed between the two time points in the gingivitis patients (*p* > 0.05). **Conclusions**: Our findings suggest that the CX3CL1–CX3CR1 axis might contribute to the inflammatory processes of periodontal diseases and may represent a treatment-responsive component of the local host response.

## 1. Introduction

Periodontal disease, a progressive and destructive inflammatory disease driven by sustained exposure to polymicrobial dysbiotic biofilm, represents a major public health concern worldwide due to its high prevalence [1,2]. The most prevalent forms of periodontal disease in humans are gingivitis and periodontitis. Gingivitis serves as a crucial risk factor and a key preliminary stage for periodontitis, and furthermore can elevate the levels of proinflammatory cytokines [3,4]. This is critical for understanding the etiopathogenesis and underlying molecular events. The molecular events that regulate periodontal inflammation also play a pivotal role in determining both the disease progression and the host’s healing response [5].

The activated host immune response to the bacterial stimuli is driven by a diverse array of chemotactic cytokines and chemokines, which collectively enhance the directed migration of immune cells to the inflamed periodontal tissues. Chemokines and their receptors are important players in the pathogenesis of periodontal disease, contributing to leukocyte infiltration, chemotaxis, angiogenesis, and the modulation of the host immune–inflammatory response [6,7].

CX3CL1, also known as fractalkine, is a chemokine of particular interest and the sole member of the CX3C chemokine subclass. It exists in both a membrane-bound form and a shed soluble form [8,9]. These two isoforms endow fractalkine with dual functionality. By binding to its specific receptor, CX3CR1, fractalkine can mediate firm cell adhesion in its membrane-bound form and act as a potent chemoattractant for immune cells in its soluble form [10,11]. This rare combination of chemotactic and adhesive properties makes the CX3CL1–CX3CR1 axis particularly relevant in inflammatory diseases where both immune cell recruitment and sustained cellular interactions are essential. Moreover, fractalkine has been proposed to play a critical role in osteoclastic activation [12]. The scientific evidence has revealed the CX3CL1–CX3CR1 axis in various important inflammatory diseases, such as atherosclerosis, rheumatoid arthritis, and chronic kidney disease [9,13,14,15]. On the other hand, the relationship between fractalkine/CX3CL1 and periodontitis has been evaluated in only a limited number of studies, and its expression has been shown in various biological samples [16,17,18,19,20]. A recent study showed that naturally occurred gingivitis had increased the GCF levels of fractalkine/CX3CL1 in comparison to experimental gingivitis [21]. To date, no studies have compared gingivitis and periodontitis in relation to this specific chemokine. Moreover, no longitudinal study is available focusing on the follow-up of the patients with periodontal disease to monitor the changes in fractalkine/CX3CL1 and its receptor CX3CR1 levels before and after periodontal therapy. Given the distinctive biological profile of fractalkine, investigating the behavior of this axis before and after treatment in patients with periodontal disease would allow us to better understand its potential involvement in the disease pathogenesis and resolution. We hypothesized that the GCF CX3CL1 and CX3CR1 levels would be elevated in patients with gingivitis and stage 3 periodontitis compared with periodontally healthy individuals, with higher levels expected in periodontitis than in gingivitis due to the greater inflammatory and destructive burden. We further hypothesized that non-surgical periodontal therapy would reduce the total GCF CX3CL1 and CX3CR1 levels in both disease groups. Based on this hypothesis, this study was designed with three objectives: First, to comparatively assess the GCF fractalkine/CX3CL1 and CX3CR1 levels among gingivitis patients, stage 3 periodontitis patients, and healthy controls. Second, to analyze the alterations in the levels of these biomarkers before and after the periodontal treatment of gingivitis and periodontitis patients. Third, to investigate the possible correlations between biochemical findings and clinical parameters, as well as the relationship between the biomarkers themselves.

## 2. Materials and Methods

### 2.1. Study Population and Clinical Evaluation

The present study included a total of 90 systemically healthy and non-smoking individuals aged 25–47 years (45 females and 45 males; age: 36.29 ± 4.77 years) from the Department of Periodontology, Faculty of Dentistry, Giresun University, Giresun, Turkey between June 2023 and December 2023. The study design was approved by the Clinical Research Ethics Committee of Ondokuz Mayis University, Samsun, Turkey (Protocol number: OMU KAEK 2022/85) and the trial was retrospectively registered at ClinicalTrials.gov (registration code: NCT07307144) on 29 December 2025. Prior to enrollment, the objectives and the procedures of this study were explained to all participants and written informed consent was obtained in compliance with the Helsinki Declaration of 1975, as revised in 2013.

A medical and dental history was obtained from each volunteer and the individuals were excluded if they had any systemic disease, had a history of smoking, were currently pregnant or lactating, had received periodontal treatment within the previous 6 months, or had used antibiotics, anti-inflammatory agents, or other medications during the same period.

The individuals with at least 20 teeth, excluding third molars, underwent a full mouth clinical and radiographic periodontal evaluation. The clinical periodontal assessment included measurements of the probing depth (PD), clinical attachment loss (CAL), gingival index (GI) [22], plaque index (PI) [23] and bleeding on probing (BOP) [24] scores. All these measurements were performed at six sites per tooth using a manual periodontal probe (Hu-Friedy, Williams Probe, Chicago, IL, USA) by a single calibrated examiner experienced in clinical research (ZPKY). For the investigator’s calibration, 10 individuals were selected randomly before the study began and examined on two separate occasions, 48 h apart. The investigator’s measurements were accepted as sufficiently reproducible if the measurements taken at baseline and at 48 h did not differ by more than 10% at the millimeter level.

Given the periodontal status, the participants were classified as healthy controls (n = 30), gingivitis (n = 30), and stage 3 periodontitis (n = 30), and matched by age and sex. Diagnoses were assigned based on the criteria outlined in the consensus report of the 2017 World Workshop on the Classification of Periodontal and Peri-Implant Diseases and Conditions [25,26]. Healthy controls included volunteers with an intact periodontium who had BOP < 10% and PD ≤ 3 mm without clinical attachment loss or radiographic sign of alveolar bone destruction. They also had no history of periodontitis. The gingivitis patients consisted of the volunteers having PD ≤ 3 mm with BOP > 30% in the entire mouth, and no clinical attachment loss or alveolar bone loss. The inclusion criteria for the generalized stage 3 periodontitis patients were interdental CAL ≥ 5 mm for at least 2 non-adjacent teeth, PD ≥ 6 mm and radiographic bone loss extending to the mid-third of the root or beyond, and they showed no more than 4 teeth lost due to periodontitis. According to the extent and distribution of the disease, the periodontitis patients had CAL ≥ 5 mm affecting 30% or more of the teeth. In addition, the grade of periodontitis has been determined as indirect evidence of the rate of progression by radiographic bone loss/age. All the patients with periodontitis were assigned to grade C, as the ratio of the greatest radiographic bone loss (%)/age was >1.0.

### 2.2. GCF Sampling

GCF sampling was performed one day after the clinical periodontal measurements in the morning hours following an overnight fast. The samples were collected from the buccal aspects of two interproximal sites of single-rooted teeth in each individual using filter paper strips (PerioPaper®; ProFlow, Inc., Amityville, NY, USA). For the healthy control group, the GCF samples were obtained from the sites with no BOP or clinical signs of inflammation. For the gingivitis group, the sites presenting BOP were selected for the sampling. For the periodontitis group, the sites with a PD of 6–7 mm accompanied by radiographic bone loss and BOP were selected. Prior to the GCF sampling, the sites were isolated using cotton rolls and any supragingival plaque was carefully removed with a sterile curette, if present, and air-dried. Then, the paper strips were inserted carefully into the gingival crevice until the feeling of mild resistance and left there for 30 s. Strips contaminated with blood or saliva were discarded. The volume of absorbed crevicular fluid per strip was measured using a calibrated electronic device (Periotron 8010, Oraflow Inc., Amityville, NY, USA), whereupon the strips were immediately transferred into an individual sterile polypropylene tube and stored at −80 °C until analysis.

### 2.3. Non-Surgical Periodontal Treatment

After the baseline GCF collection, the gingivitis and periodontitis patients underwent non-surgical periodontal treatment administered by the same investigator (Z.P.K.Y.). For the gingivitis patients, the treatment included scaling, oral hygiene education, and motivation. For the periodontitis patients, scaling and root planing procedures were performed using ultrasonic devices, manual scalers, and specific curettes on a quadrant-by-quadrant basis once a week, and was completed in four weeks. No antibiotics or adjunctive medications were administered during the treatment. All participants were instructed in oral hygiene practices, including the modified-Bass brushing technique and interdental cleaning procedures (dental floss or interproximal brushes according to the patient’s needs), with motivation and the hygiene education reinforced at each visit. Upon completion of the treatment, clinical periodontal measurements were repeated, and GCF samples were collected at 1 month and 3 months for both the gingivitis and periodontitis groups. At both time points after treatment, the GCF samples were obtained from the same sites sampled at baseline.

### 2.4. Analysis of Fractalkine/CX3CL1 and CX3CR1 Levels

On the day of the analysis, 200 μL phosphate buffered saline (pH 7.4) was added to each Eppendorf centrifuge tube containing the strips. All tubes were vortexed for 1 min and then centrifuged at 10,000× *g* for 5 min +4 °C. The levels of fractalkine and CX3CR1 in the GCF were determined by enzyme-linked immunosorbent assay (ELISA) using commercially available kits (Human Fractalkine/CX3CL1 ELISA kit, Human CX3CR1 ELISA kit, Shanghai Sunred Biotechnology Co., Ltd., Shanghai, China). The analyses were performed according to the instructions provided by the manufacturer. The absorbances were read at 450 nm by the ELISA plate reader, and the concentrations were calculated by comparing the absorbance readings of the samples with the standard curves included in each assay kit. The laboratory investigator performing the ELISA analyses was blinded to the participants’ clinical diagnosis and sampling time point throughout this study. Samples were identified only by numerical laboratory codes during the biochemical analysis.

The mean inter-assay and intra-assay coefficients of variation for fractalkine/CX3CL1 were <11% and <8%, respectively. The mean inter-assay and intra-assay coefficients of variation for CX3CR1 were <12% and <10%, respectively. The detection ranges for fractalkine/CX3CL1 and CX3CR1 were 0.2 to 30 ng/mL and 0.15 to 40 ng/mL, respectively. No measurements fell outside the assay detection range. The total amounts of fractalkine and CX3CR1 were calculated due to the dilutions and the results were expressed as total proteins in the 30 s GCF samples.

### 2.5. Statistical Analysis

The sample size calculation was based on the GCF fractalkine/CX3CL1 levels, which were considered the primary outcome variable. A power analysis was conducted to estimate the minimum required sample size, indicating that at least 25 participants in each group were necessary to achieve 90% power with an effect size of 0.96 at 0.05 statistical significance level [18]. To compensate for potential dropouts during the study period, 30 participants were included in each group.

The distribution of all variables was examined by Shapiro–Wilk normality test. Since the data were not normally distributed, intergroup comparisons of the biochemical and clinical parameters were carried out using the Kruskal–Wallis non-parametric test, followed by post hoc group comparisons with the Bonferroni-adjusted Mann–Whitney U test. The Friedman and Bonferroni-adjusted Wilcoxon tests were performed to compare the baseline and post-treatment measurements at 1 and 3 months. For the Bonferroni correction, α = 0.05/3 = 0.016 was considered to be statistically significant. Differences in sex distribution among the study groups were examined with a chi-squared test. The Spearman’s rank correlation analysis was used to assess possible associations of the GCF levels of fractalkine/CX3CL1 and CX3CR1 with the clinical parameters, as well as correlations between the biomarkers themselves. All data analyses were executed using statistical software (SPSS, v22.0, IBM, Chicago, IL, USA), and the significance level was taken as *p* < 0.05 statistically.

Additionally, for between-group comparisons, the Hodges–Lehmann median differences with 95% confidence intervals and the Cliff’s delta effect sizes were calculated. For within-group repeated comparisons, the paired median changes, percentage changes, 95% confidence intervals, Wilcoxon signed-rank *p*-values, Bonferroni-adjusted *p*-values, and paired rank-biserial effect sizes were reported. As sensitivity analyses, linear mixed-effects models were used to evaluate any longitudinal biomarker changes while accounting for repeated measurements within the participants. Models included fixed effects for group, time, and group-by-time interaction, with participant-level random intercepts. Additional models adjusted the total biomarker amounts for the GCF volume. Exploratory ROC analyses were performed to describe the baseline discriminatory performance for periodontal health versus gingivitis, gingivitis versus periodontitis, and periodontal health versus periodontitis. AUC values with 95% confidence intervals, data-derived cut-off values, sensitivity, specificity, and likelihood ratios were reported. The results of the analyses are presented in the Supplementary Materials.

## 3. Results

### 3.1. Demographics and Clinical Findings

The demographic characteristics of the study groups are presented in Table 1. There were no significant differences in age and sex distribution between the study groups (*p* > 0.05). The number of teeth was lower in the periodontitis patients than both the gingivitis patients and the healthy controls (*p* < 0.01).

The full-mouth and the sampling-site clinical findings of the study groups are presented in Table 2. The periodontitis patients exhibited significantly higher values for all periodontal parameters of the full mouth and sampling sites than healthy individuals (*p* < 0.001). The gingivitis patients had significantly higher values for all clinical periodontal variables, except for CAL, compared to the healthy controls (*p* < 0.001). The periodontitis patients had significantly higher full-mouth and sampling-sites PD and CAL values than the gingivitis patients (*p* < 0.001). The GCF volume of the periodontitis patients was also higher than that of the gingivitis patients (*p* < 0.001). Significant improvements in all these clinical parameters were observed for the full mouth as well as the sampling sites at 1 and 3 months after non-surgical periodontal therapy in the patients with gingivitis and periodontitis (*p* < 0.001).

### 3.2. Biochemical Findings

The GCF fractalkine/CX3CL1 and CX3CR1 total amounts of the study groups are outlined in Table 3. Both the gingivitis and periodontitis patients exhibited significantly increased GCF fractalkine and CX3CR1 total amounts compared to the healthy individuals (*p* < 0.001 for both). The GCF total amounts of fractalkine and CX3CR1 were also found to be higher in the periodontitis group than the gingivitis group (*p* < 0.001 for both). Significant decreases in the fractalkine and CX3CR1 levels were observed at 1 and 3 months following periodontal therapy in both the gingivitis and periodontitis patients when compared with the baseline values (*p* < 0.001 for all, Table 3 and Table 4). In the periodontitis patients, significant reductions of the GCF fractalkine and CX3CR1 levels were observed at 3 months after non-surgical periodontal treatment compared to the levels at 1 month post-treatment (*p* < 0.001 for both). By contrast, no statistically significant difference was detected in the GCF fractalkine and CX3CR1 levels between 1 and 3 months after treatment in the gingivitis patients (*p* > 0.05). The GCF levels of fractalkine and CX3CR1 were higher for both diseased groups than those of the healthy controls after treatment (*p* < 0.001, Table 3 and Table 5).

### 3.3. Correlations

Both the GCF fractalkine/CX3CL1 and CX3CR1 total amounts demonstrated significant positive correlations with the sampling site PD, CAL, and GI (*p* < 0.001 for all, Table 6). The fractalkine levels showed a strong and positive association with its receptor CX3CR1 levels in the GCF (*p* < 0.001, Table 6).

### 3.4. Diagnostic Value Findings

Exploratory ROC analyses were performed to describe the baseline discriminatory performance, and incremental-value models were used to evaluate whether CX3CL1 and CX3CR1 improved discrimination beyond the clinical periodontal parameters. These results are presented in Supplementary Tables S1 and S2.

## 4. Discussion

The current study investigated fractalkine/CX3CL1 and related receptor CX3CR1 in the GCF of patients with gingivitis and stage 3 periodontitis, with a particular focus on the alterations in their levels following non-surgical periodontal treatment. According to our findings, GCF fractalkine/CX3CL1 and CX3CR1 were significantly higher in both the gingivitis and periodontitis patients compared to the healthy individuals. These elevated levels of CX3CL1 and CX3CR1 were also found to be decreased after non-surgical periodontal treatment in both the gingivitis and periodontitis patients. In the periodontitis patients, the reduction of these molecules was significantly greater at the 3 month follow-up compared to the levels observed at the 1 month follow-up after therapy. Moreover, strong correlations were observed between the GCF levels of CX3CL1 and CX3CR1 and the sampling site clinical parameters, PD, CAL, and GI. The current data support the involvement of CX3CL1 and CX3CR1 in the inflammatory processes associated with periodontal diseases.

Biochemical molecules of host or microbial origin are increasingly recognized as valuable tools for detecting pathogenic processes and for monitoring therapeutic outcomes. Given its site-specific nature, GCF serves as an ideal medium for investigating the local molecular events that drive the onset and progression of periodontal inflammation [27,28]. Moreover, an assessment of the GCF composition before and after therapy enables the evaluation of biomarker dynamics and responsiveness. Accordingly, GCF was selected as the biological fluid for analysis in this study. The data were presented as the total amount of a constituent in the GCF rather than its concentration, which is the more appropriate and reliable indicator to identify the GCF constituents related to periodontal disease.

While the role of the fractalkine/CX3CL1–CX3CR1 axis in autoimmune disorders, particularly rheumatoid arthritis, is well characterized, little is known about its involvement in the inflammatory processes of periodontal diseases [8,29]. The expression of the chemokine CX3CL1 in different biological samples has recently been demonstrated in periodontal disease [16,17,18,19,20,21,30]. Yılmaz et al. [16] reported greater salivary CX3CL1 levels in rheumatoid arthritis patients than in periodontitis patients with or without rheumatoid arthritis and in healthy individuals. They also found higher serum CX3CL1 levels in rheumatoid arthritis patients with periodontitis compared to the other study groups. Similarly, Panezai et al. [17] demonstrated higher serum CX3CL1 levels in rheumatoid arthritis patients with chronic periodontitis compared to the periodontitis, rheumatoid arthritis, and control groups. In another study, serum levels of CX3CL1 were lower in periodontitis patients than periodontally healthy ones [18]. Kawamoto et al. [19] showed elevated levels of both CX3CL1 and CX3CR1 in the saliva of periodontitis patients compared to controls; however, this increase was not significant. Conversely, Harbood and Abbas [30] reported significantly higher fractalkine levels in the saliva of periodontitis patients than in healthy controls. Accordingly, the existing literature reporting salivary and serum fractalkine levels presents conflicting results. This discrepancy may be associated with the possibility that the severity of local periodontal inflammation is insufficient to elicit a measurable systemic response and, additionally, to the fact that saliva reflects the entire oral cavity, which may obscure site-specific inflammatory changes and lead to inconsistent findings. On the other hand, Hosokawa et al. [20] demonstrated robust CX3CL1 mRNA expression in gingival tissue samples obtained from individuals with periodontitis. Furthermore, a recent study analyzing GCF CX3CL1 and CX3CR1 levels in individuals with periodontitis reported increased concentrations of this axis in patients with stage 3 grade B periodontitis [18]. Consistent with these findings, our study also revealed a significant elevation of the GCF CX3CL1 and CX3CR1 levels in individuals with stage 3 grade C periodontitis. In individuals with gingivitis, only a single study to date has investigated fractalkine levels, reporting that naturally occurring gingivitis is associated with higher GCF levels of CX3CL1 compared with periodontally healthy individuals [21]. In agreement with this observation, our study also identified substantially elevated levels of both CX3CL1 and its receptor CX3CR1 in the gingivitis group relative to healthy controls. Moreover, as a novel contribution, our comparative analysis revealed higher GCF CX3CL1 and CX3CR1 levels in the patients with periodontitis than those with gingivitis. So, we can speculate that the infiltration of specific leukocytes to the damaged area may contribute microbial dysbiosis, inflammation, alveolar bone destruction, and the progression of periodontal disease in the presence and persistence of periodontopathogens. Collectively, these results support our hypothesis and suggest that fractalkine and its receptor may reflect the magnitude of the local inflammatory response in periodontal disease.

Meanwhile, the current study is the first to evaluate the CX3CL1–CX3CR1 axis in gingivitis and periodontitis before and after periodontal therapy. In our research, a significant reduction in the GCF CX3CL1 and CX3CR1 levels was observed at 1 month after periodontal treatment in both the gingivitis and periodontitis groups. Furthermore, in the periodontitis patients, these reductions became even more pronounced at 3 months after treatment when compared with the 1 month results. No additional decrease between 1 and 3 months was detected in the gingivitis group. This pattern is consistent with the reversible nature of gingival inflammation, in which short-term resolution typically occurs following the elimination of plaque-induced stimuli. On the other hand, it should be considered that the different treatment intensity and duration may influence the temporal pattern of biomarker reduction, when comparing the post-treatment outcomes between gingivitis and periodontitis groups. By contrast, the persistence of the inflammatory burden in periodontitis makes the return to healthy biomarker levels more challenging; thus, the continued decline in fractalkine and CX3CR1 levels from 1 to 3 months may reflect a gradual resolution of deeper and more chronic inflammatory processes. Despite these reductions, the post-treatment total biomarker levels, particularly in the periodontitis group, remained higher than the healthy baseline levels. This may reflect the ongoing host response activity or incomplete biological resolution. These observations suggest that the CX3CL1-CX3CR1 axis may serve as a biological indicator of local inflammatory burden.

Exploratory ROC analyses demonstrated that the baseline CX3CL1 and CX3CR1 levels were capable of discriminating between periodontal health and inflammatory periodontal conditions. However, the highest discriminatory performance was observed for contrasts involving periodontal health and stage 3 periodontitis, which may be susceptible to spectrum effects. Furthermore, exploratory incremental value analyses indicated that conventional clinical periodontal parameters and GCF volume already provided near-perfect discrimination between the study groups. The addition of CX3CL1 and CX3CR1 did not meaningfully improve the cross-validated model performance. Therefore, the present findings do not support the incremental diagnostic utility of these biomarkers beyond a conventional periodontal assessment. Instead, CX3CL1 and CX3CR1 should be interpreted as treatment-responsive inflammatory markers associated with local periodontal inflammatory status.

These exploratory analyses should also be interpreted cautiously because the periodontal group classification was based on conventional clinical periodontal parameters, introducing a degree of partial circularity into the discrimination models. Consequently, the near-perfect AUC values observed in this study should not be interpreted as externally validated estimates of diagnostic accuracy and require confirmation in broader, less selected populations. In addition, although conventional clinical parameters remain essential for periodontal diagnosis and treatment monitoring, they primarily reflect accumulated tissue destruction and clinical manifestations and cumulative consequences of disease. Biomarkers detected in GCF may provide complementary information regarding the underlying biological activity within periodontal tissues. In the present study, the CX3CL1 and CX3CR1 levels increased progressively from periodontal health to gingivitis and periodontitis and decreased significantly following non-surgical periodontal therapy. Therefore, the potential value of CX3CL1 and CX3CR1 may not lie in replacing established clinical measures but rather in improving our understanding of local inflammatory processes and, potentially, contributing to future biomarker-based risk assessment strategies.

As another key finding of our study, positive correlations were observed between the GCF CX3CL1 and CX3CR1 levels and clinical periodontal parameters, including PD, CAL, and GI. These findings suggest an association between the fractalkine-CX3CR1 axis and the periodontal inflammatory status. However, because the correlation analyses were performed using pooled data from all study groups, the observed associations may partly reflect differences between periodontal health, gingivitis, and periodontitis rather than within-group relationships. Nevertheless, we also demonstrated a positive association between the GCF CX3CL1 and CX3CR1 levels, reflecting their coordinated involvement in periodontal inflammation. This finding aligns with the known biological mechanism whereby fractalkine-CX3CR1 interactions mediate leukocyte capture and adhesion to endothelial cells, enhance integrin activation and proinflammatory cytokine production, and promote leukocyte transmigration into inflamed tissues [29]. Collectively, these observations support a potential role of this chemokine–receptor axis in periodontal inflammatory processes and tissue damage.

It is also important to note that osteoblasts have been identified as a source of CX3CL1 in inflamed bone and joint tissues, while, on the other hand, osteoclast precursor cells express its receptor, CX3CR1 [12]. This ligand–receptor interaction has been shown to facilitate the chemotactic recruitment of osteoclast precursors, to promote their firm adhesion to osteoblasts, and ultimately to support their differentiation into mature osteoclasts. CX3CL1 acts as a potent chemoattractant for preosteoclasts migrating through the osteoblast layer, thereby enhancing osteoclastogenesis [31]. Such CX3CL1-mediated osteoclast activation raises the possibility that this axis may contribute to alveolar bone resorption and periodontal tissue destruction. Within the scope of our findings, the significant reduction of the GCF CX3CL1-CX3CR1 levels following periodontal therapy in periodontitis patients in parallel to clinical improvement may indicate the effective control of alveolar bone loss. Notably, the more pronounced decrease detected at 3 months relative to 1 month after treatment in the periodontitis patients may reflect a progressive downregulation of CX3CL1- and CX3CR1-mediated osteoclast activation, which may be indicative of a gradual shift toward improved regulation of alveolar bone turnover alongside clinical improvement. Considering the potential involvement of this chemokine–receptor axis in both immunoinflammatory responses and tissue breakdown in periodontal disease, therapeutic modulation of the CX3CL1–CX3CR1 pathway may represent a promising treatment strategy, as has been previously proposed for rheumatoid arthritis [29,32]. Nevertheless, the present study measured soluble GCF levels only. Membrane-bound CX3CL1 expression, tissue-level CX3CR1 expression, osteoclast activity, and bone turnover markers were not assessed. Therefore, any link between the CX3CL1-CX3CR1 axis and alveolar bone resorption in the present context should be considered hypothesis-generating.

The present study has several limitations. First, the study population consisted of relatively young, systemically healthy, non-smoking individuals recruited from a single center, which may limit the generalizability of the results to broader populations. In addition, only patients diagnosed with stage 3 grade C periodontitis were included in the periodontitis group. Therefore, the observed associations between the CX3CL1/CX3CR1 levels and periodontal inflammatory status may not be directly applicable to patients with other stages or grades of periodontitis, smokers, older individuals, or those with systemic comorbidities. Second, the follow-up period was limited to 3 months and does not permit an evaluation of long-term biomarker dynamics. Third, an untreated disease control group was not included, and healthy controls were sampled only at baseline. Therefore, treatment-related changes cannot be fully separated from regression to the mean, plaque reduction, oral hygiene reinforcement, or natural short-term fluctuation. Fourth, microbiological analyses were not performed, preventing an assessment of the relationship between CX3CL1/CX3CR1 levels and periodontal microbial dysbiosis. An additional limitation of the present study is that registration at ClinicalTrials.gov was performed retrospectively. Future multicenter studies involving more diverse patient populations are needed to confirm the generalizability of these findings. Also, a comprehensive analysis of the molecular mechanisms at the expression level of this chemokine and its receptor, together with microbiological assessments of periodontal tissue responses, would be essential to clarify the fractalkine’s roles in periodontal diseases.

## 5. Conclusions

The present study demonstrated for the first time the impact of non-surgical periodontal therapy on GCF fractalkine/CX3CL1 and its receptor CX3CR1 levels in individuals with gingivitis and periodontitis. The marked elevation of these molecules from periodontal health to disease, followed by their gradual and significant reduction after treatment, suggests that the fractalkine–CX3CR1 axis may be involved in periodontal inflammatory processes and may represent a treatment-responsive component of the local host response. Therefore, CX3CL1 and CX3CR1 may have potential utility as exploratory biomarkers of the local inflammatory burden in periodontal disease. Further prospective, multicenter, controlled, and large-scale studies incorporating microbiological and longitudinal assessments are required to better define the potential clinical relevance of the CX3CL1–CX3CR1 axis in periodontal disease.

## Figures and Tables

**Table 1:** Demographic Characteristics of the Study Groups.

	Healthy (n = 30)	Gingivitis (n = 30)	Periodontitis (n = 30)
**Demographic variables**			
Age (years)	37 (25–42)	36 (28–44)	37 (31–47)
Sex (F/M)	16/14	14/16	15/15
Number of teeth	28 (26–28)	28 (24–28)	26 (24–28) *

Data are expressed as median (minimum-maximum). * Significantly different from the healthy and gingivitis groups (*p* < 0.001).

**Table 2:** Clinical Periodontal Measurements of the Study Groups.

Periodontal Parameters	Time	Healthy (n = 30)	Gingivitis (n = 30)	Periodontitis (n = 30)
***Full-mouth***				
	Baseline	1.79 (1.62–1.99)	2.59 (2.50–2.67) *	4.62 (4.46–4.88) *^†^
PD (mm)	1 month		2.08 (1.90–2.27) ^‡^	2.82 (2.70–3.04) ^‡^
	3 months		2.08 (1.88–2.25) ^‡^	2.74 (2.55–2.97) ^‡^
	Baseline	0.00 (0.00–0.00)	0.00 (0.00–0.00)	5.51 (5.27–5.88) * ^†^
CAL (mm)	1 month		0.00 (0.00–0.00)	3.93 (3.05–4.79) ^‡^
	3 months		0.00 (0.00–0.00)	3.93 (3.58–4.25) ^‡^
	Baseline	0.12 (0.03–0.22)	2.35 (2.09–2.60) *	2.60 (2.32–2.74) *
GI	1 month		0.36 (0.25–0.69) ^‡^	0.52 (0.36–0.68) ^‡^
	3 months		0.35 (0.24–0.55) ^‡^	0.38 (0.28–0.48) ^‡§^
	Baseline	0.28 (0.19–0.44)	1.84 (1.52–2.17) *	1.82 (1.60–2.06) *
PI	1 month		0.66 (0.43–0.81) ^‡^	0.78 (0.62–0.90) ^‡^
	3 months		0.66 (0.44–0.80) ^‡^	0.69 (0.50–0.89) ^‡^
	Baseline	0.00 (0.00–0.44)	86.42 (76.94–92.78) *	85.52 (80.27–92.13) *
BOP (%)	1 month		5.57 (2.11–8.86) ^‡^	9.43 (7.95–12.05) ^‡^
	3 months		5.52 (3.75–8.05) ^‡^	6.80 (5.77–8.45) ^‡§^
***Sampling site***				
	Baseline	2.00 (1.50–2.00)	3.00 (2.62–3.00) *	6.50 (6.00–7.00) *^†^
PD (mm)	1 month		2.00 (2.00–2.50) ^‡^	3.00 (3.00–3.00) ^‡^
	3 months		2.50 (2.00–2.50) ^‡^	3.00 (3.00–3.00) ^‡§^
	Baseline	0.00 (0.00–0.00)	0.00 (0.00–0.00)	7.00 (6.62–7.50) *^†^
CAL (mm)	1 month		0.00 (0.00–0.00)	4.00 (3.50–4.50) ^‡^
	3 months		0.00 (0.00–0.00)	4.00 (3.50–4.00) ^‡^
	Baseline	0.00 (0.00–0.00)	2.50 (2.50–3.00) *	2.00 (2.00–2.00) *
GI	1 month		0.00 (0.00–0.00) ^‡^	0.00 (0.00–0.00) ^‡^
	3 months		0.00 (0.00–0.38) ^‡^	0.00 (0.00–0.00) ^‡^
	Baseline	0.00 (0.00–0.50)	2.25 (1.62–2.50) *	2.00 (2.00–2.50) *
PI	1 month		0.00 (0.00–0.38) ^‡^	0.00 (0.00–0.50) ^‡^
	3 months		0.00 (0.00–0.50) ^‡^	0.00 (0.00–0.50) ^‡^
	Baseline	0.00 (0.00–0.00)	100.00 (100.00–100.00) *	100.00 (100.00–100.00) *
BOP (%)	1 month		0.00 (0.00–0.00) ^‡^	0.00 (0.00–0.00) ^‡^
	3 months		0.00 (0.00–0.00) ^‡^	0.00 (0.00–0.00) ^‡^
	Baseline	0.08 (0.06–0.11)	0.55 (0.45–0.64) *	0.76 (0.69–0.84) *^†^
GCF volume (µL)	1 month		0.11 (0.08–0.15) ^‡^	0.19 (0.15–0.24) ^‡^
	3 months		0.12 (0.09–0.16) ^‡^	0.16 (0.12–0.21) ^‡§^

PD: probing depth; CAL: clinical attachment loss; GI: gingival index; PI: plaque index; BOP: bleeding on probing; GCF: gingival crevicular fluid. Data are expressed as median (interquartile range). * Significantly higher than the healthy controls (*p* < 0.001). ^†^ Significantly higher than the gingivitis group (*p* < 0.001). ^‡^ Significant difference from the baseline value in the group (*p* < 0.001). ^§^ Significant difference between the 1-month and 3-month values in the group (*p* < 0.001).

**Table 3:** Fractalkine and CX3CR1 Levels in GCF of the Study Groups.

	Healthy (n = 30)	Gingivitis (n = 30)	Periodontitis (n = 30)
**Fractalkine** **(ng/30-s samples)**			
Baseline	0.12 (0.65–0.17)	1.02 (0.73–1.24) *	2.32 (1.95–2.80) *^†^
1 month		0.21 (0.19–0.25) ^‡^*	0.79 (0.65–1.01) ^‡^*
3 months		0.20 (0.17–0.24) ^‡^*	0.46 (0.40–0.69) ^‡§^*
**CX3CR1** **(ng/30-s samples)**			
Baseline	0.19 (0.14–0.24)	1.75 (1.38–2.11) *	3.71 (3.22–4.29) *^†^
1 month		0.32 (0.18–0.39) ^‡^*	1.23 (1.04–1.79) ^‡^*
3 months		0.32 (0.18–0.41) ^‡^*	0.99 (0.81–1.14) ^‡§^*

Data are expressed as median (interquartile range). * Significantly higher than the healthy controls (*p* < 0.001). ^†^ Significantly higher than the gingivitis group (*p* < 0.001). ^‡^ Significant difference from the baseline value in the group (*p* < 0.001). ^§^ Significant difference between the 1-month and 3-month values in the group (*p* < 0.001).

**Table 4:** Magnitude of Treatment-related Changes in Total GCF CX3CL1 and CX3CR1 Amounts.

Biomarker	Group	Comparison	Median Absolute Change (95% CI)	Median % Change	Effect Size (r)	Adjusted *p*-Value
CX3CL1	Gingivitis	Baseline to	−0.72 (−0.92 to −0.55)	−76.5%	−1.000	<0.001
		1 month				
CX3CL1	Gingivitis	Baseline to	−0.74 (−0.93 to −0.58)	−77.5%	−1.000	<0.001
		3 months				
CX3CL1	Periodontitis	Baseline to	−1.59 (−1.86 to −1.36)	−63.8%	−1.000	<0.001
		1 month				
CX3CL1	Periodontitis	Baseline to	−1.86 (−2.07 to −1.64)	−78.0%	−1.000	<0.001
		3 months				
CX3CR1	Gingivitis	Baseline to	−1.44 (−1.82 to −1.04)	−82.2%	−1.000	<0.001
		1 month				
CX3CR1	Gingivitis	Baseline to	−1.40 (−1.78 to −1.01)	−81.6%	−1.000	<0.001
		3 months				
CX3CR1	Periodontitis	Baseline to	−2.42 (−2.80 to −2.05)	−64.2%	−1.000	<0.001
		1 month				
CX3CR1	Periodontitis	Baseline to	−2.73 (−3.04 to −2.36)	−74.7%	−1.000	<0.001
		3 months				

Values represent median within-subject changes from the baseline. Effect sizes are reported as paired rank-biserial correlation coefficients derived from Wilcoxon signed-rank tests. Confidence intervals represent bootstrap 95% confidence intervals for the median change. *p*-values were Bonferroni-adjusted for multiple comparisons.

**Table 5:** Comparison of Post-treatment Biomarker Levels with Healthy Values.

Biomarker	Comparison	Median Ratio vs. Healthy	Hodges–Lehmann Difference (95% CI)	Cliff’s δ (95% CI)	Mann–Whitney *p*	Bonferroni-Adjusted *p*
CX3CL1	Gingivitis 1 month vs. Healthy baseline	1.70	0.10 (0.07–0.14)	0.77 (0.58–0.92)	<0.001	<0.001
CX3CL1	Gingivitis 3 months vs. Healthy baseline	1.57	0.09 (0.05–0.12)	0.70 (0.48–0.88)	<0.001	<0.001
CX3CL1	Periodontitis 1 month vs. Healthy baseline	6.20	0.68 (0.58–0.80)	1.00 (1.00–1.00)	<0.001	<0.001
CX3CL1	Periodontitis 3 months vs. Healthy baseline	3.65	0.36 (0.31–0.47)	0.99 (0.98–1.00)	<0.001	<0.001
CX3CR1	Gingivitis 1 month vs. Healthy baseline	1.64	0.10 (0.03–0.17)	0.52 (0.26–0.75)	<0.001	0.001
CX3CR1	Gingivitis 3 months vs. Healthy baseline	1.63	0.11 (0.04–0.18)	0.55 (0.32–0.77)	<0.001	<0.001
CX3CR1	Periodontitis 1 month vs. Healthy baseline	6.35	1.07 (0.93–1.42)	1.00 (1.00–1.00)	<0.001	<0.001
CX3CR1	Periodontitis 3 months vs. Healthy baseline	5.10	0.79 (0.68–0.88)	1.00 (1.00–1.00)	<0.001	<0.001

Post-treatment biomarker levels in the gingivitis and periodontitis groups at 1 month and 3 months were compared with baseline values of the healthy control group. The results are presented as Hodges–Lehmann median differences with 95% confidence intervals (CI), Cliff’s delta effect sizes with 95% CI, Mann–Whitney U test *p*-values, Bonferroni-adjusted *p*-values, and median ratios relative to healthy baseline levels. Median ratios >1 indicate biomarker levels remaining elevated compared with healthy controls.

**Table 6:** Spearman Rank’s Correlations of GCF Fractalkine and CX3CR1 with Sampling Site Clinical Parameters and with Each Other in All Groups.

	PD (mm)	CAL (mm)	GI	GCF Fractalkine (ng/30-s Samples)	GCF CX3CR1 (ng/30-s Samples)
GCF Fractalkine (ng/30-s samples)	0.948 *	0.821 *	0.586 *	1.000	0.981 *
GCF CX3CR1 (ng/30-s samples)	0.950 *	0.825 *	0.594 *	0.981 *	1.000

* Statistically significant (*p* < 0.001).

## Data Availability

The data for this study are available from the corresponding author upon reasonable request under ethical and legal restrictions related to patient confidentiality.

## Supplementary Materials

The following supporting information can be downloaded at: https://www.mdpi.com/article/10.3390/jcm15134922/s1, Table S1: Exploratory incremental-value analyses for baseline group discrimination; Table S2: Exploratory ROC analyses of baseline total GCF CX3CL1 and CX3CR1 levels; Table S3: Baseline between-group comparisons in clinical periodontal parameters; Table S4: Treatment-induced changes in clinical periodontal parameters; Table S5: Linear mixed-effects sensitivity analysis for CX3CL1 total amount and GCF volume-adjusted CX3CL1 total amount; Table S6: Comparison of estimated baseline-to-3-month changes in CX3CL1 and CX3CR1 total amounts before and after adjustment for GCF volume.

## Abbreviations

The following abbreviations are used in this manuscript:

GCF	Gingival crevicular fluid
PD	Probing depth
CAL	Clinical attachment loss
GI	Gingival index
PI	Plaque index
BOP	Bleeding on probing
CX3CL1	C-X3-C motif chemokine ligand 1
CX3CR1	C-X3-C motif chemokine receptor 1
ELISA	Enzyme-linked immunosorbent assay
