# Supplementary material for: Fractalkine and CX3CR1 Levels in Gingivitis and Stage 3 Periodontitis Patients Following Non-Surgical Periodontal Therapy: A Prospective Clinical Study
Source: J Clin Med. 2026 Jun 24;15(13):4922. doi: 10.3390/jcm15134922 (PMC13362455; doi:10.3390/jcm15134922)
Supplement: Supplementary file 1 [file jcm-15-04922-s001.zip › jcm-4336124-supplementary.pdf]

Supplementary Tables

Table S1. Exploratory incremental-value analyses for baseline group discrimination.

Comparison	Clinical + GCF CV AUC	Clinical + GCF + Total biomarker CV AUC	ΔAUC	Clinical + GCF + Concentration biomarker CV AUC	ΔAUC
Healthy vs Gingivitis	1.000	1.000	0.000	1.000	0.000
Gingivitis vs Periodontitis	1.000	1.000	0.000	1.000	0.000
Healthy vs Periodontitis	1.000	1.000	0.000	1.000	0.000

AUC, area under the receiver operating characteristic curve; CV, cross-validated. Total biomarker models included CX3CL1 and CX3CR1 total amounts, whereas concentration biomarker models included volume-normalized CX3CL1 and CX3CR1 concentrations. ΔAUC values were calculated relative to the Clinical + GCF volume model.

Table S2. Exploratory ROC analyses of baseline total GCF CX3CL1 and CX3CR1 levels

Comparison	Biomarker	AUC	95% CI	Cut-off	Sensitivity (%)	Specificity (%)
Healthy vs Gingivitis	CX3CL1	1.000	1.000–1.000	0.517	100.0	100.0
Healthy vs Gingivitis	CX3CR1	1.000	1.000–1.000	0.873	100.0	100.0
Gingivitis vs Periodontitis	CX3CL1	1.000	1.000–1.000	1.604	100.0	100.0
Gingivitis vs Periodontitis	CX3CR1	1.000	1.000–1.000	2.979	100.0	100.0
Healthy vs Periodontitis	CX3CL1	1.000	1.000–1.000	1.604	100.0	100.0
Healthy vs Periodontitis	CX3CR1	1.000	1.000–1.000	2.979	100.0	100.0

AUC, area under the receiver operating characteristic curve; CI, confidence interval. Cut-off values were determined using the Youden index. ROC analyses were performed using baseline measurements only and should be considered exploratory.

Table S3. Baseline between-group comparisons in clinical periodontal parameters.

Variable	Comparison	Hodges–Lehmann Difference	95% CI	Cliff's δ	p-value
PD (mm)	Healthy vs Gingivitis	-0.79	-0.92 to -0.66	-0.979	<0.001
	Healthy vs Periodontitis	-2.88	-3.03 to -2.75	-1.000	<0.001
	Gingivitis vs Periodontitis	-2.08	-2.23 to -1.94	-1.000	<0.001
CAL (mm)	Healthy vs Gingivitis	0.00	0.00 to 0.00	0.000	1.000
	Healthy vs Periodontitis	-5.52	-5.79 to -5.43	-1.000	<0.001
	Gingivitis vs Periodontitis	-5.52	-5.79 to -5.43	-1.000	<0.001
GI	Healthy vs Gingivitis	-2.22	-2.36 to -2.05	-1.000	<0.001
	Healthy vs Periodontitis	-2.41	-2.53 to -2.27	-1.000	<0.001
	Gingivitis vs Periodontitis	-0.16	-0.35 to 0.01	-0.278	0.065
PI	Healthy vs Gingivitis	-1.52	-1.72 to -1.33	-1.000	<0.001
	Healthy vs Periodontitis	-1.50	-1.68 to -1.35	-1.000	<0.001
	Gingivitis vs Periodontitis	0.04	-0.18 to 0.22	0.049	0.751
BOP (%)	Healthy vs Gingivitis	-86.33	-90.47 to -79.62	-1.000	<0.001
	Healthy vs Periodontitis	-84.93	-88.12 to -80.66	-1.000	<0.001
	Gingivitis vs Periodontitis	-0.67	-5.76 to 4.36	-0.061	0.690
GCF volume (μL)	Healthy vs Gingivitis	-0.46	-0.52 to -0.39	-1.000	<0.001
	Healthy vs Periodontitis	-0.68	-0.72 to -0.63	-1.000	<0.001
	Gingivitis vs Periodontitis	-0.22	-0.28 to -0.16	-0.830	<0.001

Between-group comparisons were performed using the Mann–Whitney U test. Hodges–Lehmann estimates represent median differences between groups with corresponding 95% confidence intervals (CI). Effect sizes were calculated using Cliff's delta (δ).

Table S4. Treatment-induced changes in clinical periodontal parameters.

Variable	Group	Median Change	95% CI	Paired Rank-Biserial Effect Size	p-value
PD (mm)	Gingivitis	-0.52	-0.68 to -0.42	-0.983	<0.001
	Periodontitis	-1.88	-2.08 to -1.73	-1.000	<0.001
CAL (mm)	Gingivitis	0.00	0.00 to 0.00	0.000	1.000
	Periodontitis	-1.64	-1.82 to -1.37	-1.000	<0.001
GI	Gingivitis	-1.89	-2.14 to -1.72	-1.000	<0.001
	Periodontitis	-2.21	-2.31 to -1.88	-1.000	<0.001
PI	Gingivitis	-1.32	-1.47 to -1.04	-1.000	<0.001
	Periodontitis	-1.11	-1.33 to -0.97	-0.996	<0.001
BOP (%)	Gingivitis	-79.68	-85.65 to -74.05	-1.000	<0.001
	Periodontitis	-78.26	-83.04 to -72.97	-1.000	<0.001
GCF volume (μL)	Gingivitis	-0.44	-0.48 to -0.37	-1.000	<0.001
	Periodontitis	-0.61	-0.66 to -0.55	-1.000	<0.001

Within-group comparisons were performed using the Wilcoxon signed-rank test. Median change was calculated as the difference between baseline and post-treatment values. Confidence intervals (95% CI) were estimated using the Hodges–Lehmann approach for paired samples. Effect sizes were expressed as paired rank-biserial correlation coefficients.

Table S5. Linear mixed-effects sensitivity analysis for CX3CL1 total amount and GCF volume-adjusted CX3CL1 total amount.

Contrast	Estimate Ratio (95% CI)	% Change	p-value
**Unadjusted model (total CX3CL1 amount)**			
Gingivitis: 3 months vs baseline	0.216 (0.196–0.238)	−78.4%	<0.001
Periodontitis: 3 months vs baseline	0.214 (0.194–0.236)	−78.6%	<0.001
Periodontitis vs Gingivitis at baseline	2.442 (2.096–2.845)	+144.2%	<0.001
Periodontitis vs Gingivitis at 3 months	2.415 (2.073–2.814)	+141.5%	<0.001
Group × time interaction (baseline to 1 month)	1.428 (1.244–1.639)	+42.8%	<0.001
Group × time interaction (baseline to 3 months)	1.012 (0.881–1.163)	+1.2%	0.863
**GCF volume-adjusted model**			
Gingivitis: 3 months vs baseline	0.248 (0.195–0.316)	−75.2%	<0.001
Periodontitis: 3 months vs baseline	0.248 (0.192–0.320)	−75.2%	<0.001
Periodontitis vs Gingivitis at baseline	2.363 (2.017–2.769)	+136.3%	<0.001
Periodontitis vs Gingivitis at 3 months	2.358 (2.021–2.752)	+135.8%	<0.001
Group × time interaction (baseline to 1 month)	1.398 (1.211–1.614)	+39.8%	<0.001
Group × time interaction (baseline to 3 months)	0.998 (0.863–1.154)	−0.2%	0.979

Linear mixed-effects models included fixed effects for group, time, and group-by-time interaction, with participant-level random intercepts.

Table S6. Comparison of estimated baseline-to-3-month changes in CX3CL1 and CX3CR1 total amounts before and after adjustment for GCF volume.

Biomarker	Group	Total amount model	GCF volume-adjusted model
CX3CL1	Gingivitis	−78.4%, p < 0.001	−75.2%, p < 0.001
CX3CL1	Periodontitis	−78.6%, p < 0.001	−75.2%, p < 0.001
CX3CR1	Gingivitis	−82.1%, p < 0.001	−72.5%, p < 0.001
CX3CR1	Periodontitis	−74.6%, p < 0.001	−60.0%, p < 0.001

Data are presented as model-estimated percentage changes from baseline to 3 months. Significant reductions in both biomarkers remained after adjustment for GCF volume.
